# Ribotype 078 *Clostridium difficile* infection incidence in Dutch hospitals is not associated with provincial pig farming: Results from a national sentinel surveillance, 2009-2015

**DOI:** 10.1371/journal.pone.0189183

**Published:** 2017-12-29

**Authors:** Sofie M. van Dorp, Sabine C. de Greeff, Céline Harmanus, Ingrid M. J. G. Sanders, Olaf M. Dekkers, Cornelis W. Knetsch, Greetje A. Kampinga, Daan W. Notermans, Ed J. Kuijper

**Affiliations:** 1 Department of Medical Microbiology, Leiden University Medical Center, Leiden, the Netherlands; 2 Centre for Infectious Disease Control, the National Institute for Public Health and the Environment (RIVM), Bilthoven, the Netherlands; 3 Department of Clinical Epidemiology and Endocrinology and Metabolic Diseases, Leiden University Medical Center, Leiden, the Netherlands; 4 Department of Medical Microbiology, University of Groningen, University Medical Center Groningen, Groningen, the Netherlands; Cleveland Clinic, UNITED STATES

## Abstract

**Background:**

It has been suggested that the high incidence of ribotype 078 *Clostridium difficile* infections (CDI) in the Netherlands is related to pig farming.

**Methods:**

We used data of hospitalised CDI patients (>2yrs of age) diagnosed between May 2009 and May 2015 in 26 hospitals participating in a national sentinel surveillance. We compared clinical and geographical characteristics of 078 CDI to other CDI. We investigated the association between 078 CDI incidence and four indicators of pig farming (piglet, pig, piglet farm and pig farm density) by mixed-effects Poisson regression. We used a space-time permutation model to search for community-onset 078 CDI clusters (using SaTScan).

**Results:**

A total of 4,691 CDI were identified. Ribotype 078 was isolated in 493 of 3,756 patients (13.1%) including a typing result. These patients had slightly higher community-onset disease and a 35% increase of 30-day mortality compared to non-078 CDI patients. The pooled overall and 078 incidence rates were 2.82 (95% CI, 2.42–3.29) and 0.26 (95% CI, 0.21–0.31) CDI per 10,000 patients-days respectively. Hospital 078 CDI incidence was not associated with provincial pig (IRR, 0.98; 95% CI, 0.89–1.08), piglet (IRR, 0.95; 95% CI, 0.75–1.19), pig farm (IRR, 1.08; 95% CI, 0.84–1.39), or piglet farm density (IRR, 1.00; 95% CI, 0.56–1.79). No clusters of community-onset ribotype 078 CDI were found.

**Conclusions:**

Our results do not indicate that the ribotype 078 CDI incidence in hospitals is related to pig (farm) or piglet (farm) density. However, transmission beyond provincial borders or in non-hospitalised patients cannot be excluded.

## Introduction

The Gram-positive spore-forming bacterium *Clostridium difficile* emerged as an important cause of infectious diarrhoea and diarrhoeic outbreaks in hospitals in the Netherlands [1]. Hospitalised patients are considered to be primarily infected by other *C*. *difficile* infection (CDI) patients, possibly mediated through healthcare personnel or the hospital environment [2]. Yet, in at least 45% of the cases the source of infection is unknown [3]. Animals might be an alternative source of *C*. *difficile* infection [4, 5]. Several animal species are colonized by similar *C*. *difficile* subtypes as found in humans [4]. More in-depth genomic studies have suggested *C*. *difficile* transmission between pigs and humans of ribotype 014 in Australia [6] and ribotype 078 in the Netherlands [7]. Ribotype 078 is the predominant ribotype in pigs and piglets in the Netherlands [5, 8]. About 50%-80% of piglets are colonised by *C*. *difficile* and can develop disease [8–10]. At slaughter age, 1%-9% of pigs are positive [10, 11]. *C*. *difficile* spores can subsequently contaminate farm environments and pig-derived manure [12, 13] and can be found in meat [4, 5]. Considering the fact that 12 million pigs coexist with nearly 17 million inhabitants in the Netherlands [14], the impact of pig-farming on ribotype 078 transmission can be significant. Pig farming is concentrated in specific provinces in the Netherlands.

Since mid-2006, ribotype 078 is one of the most common ribotypes to cause CDI in hospitalised patients in the Netherlands [1]. Ribotype 078 appeared to be associated to community-acquired disease [15, 16] and more abundant in areas of concentrated pig farming in 2005–2008 [16]. In other European regions with high ribotype 078 rates, such as Northern Ireland and Scotland, the link between ribotype 078 and pig-farming was not extensively investigated [17, 18].

We hypothesise that if *C*. *difficile* ribotype 078 shedding by pigs and/or piglets leads to enhanced regional human exposure, we would find a relation between pig farming and the ribotype 078 CDI incidence in our country. In the present study, we use data of a national *C*. *difficile* infection sentinel surveillance in the Netherlands (May 2009–May 2015). We investigate the association between hospital incidence rates of ribotype 078 CDI and pig(let) density and pig(let) farm density at a provincial level. Second, we compare clinical characteristics of ribotype 078 compared to other CDI. Third, we use a space-time permutation model to identify the location of clusters of community-onset 078 CDI followed by hospitalisation.

## Methods

### Sentinel surveillance system

**Fig 1:**
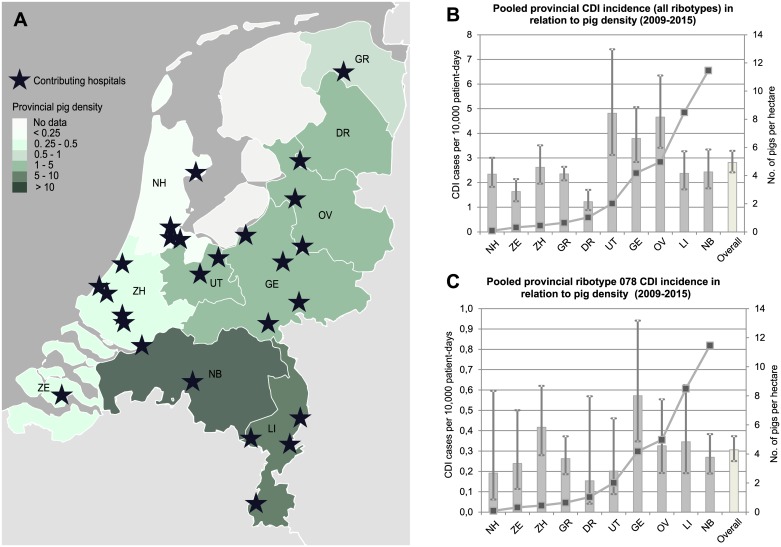
Location of sentinel surveillance hospitals and pooled CDI incidence rates in relation to average provincial pig density (no. of pigs per hectare) in 2009–2015. (a) Locations of sentinel surveillance hospitals (n = 26) and the average provincial pig density per hectare (Source of map: ArcGis, Environmental Systems Research Institutes, Inc. Redlands CA), (b) Pooled provincial CDI (all ribotypes) incidence rates and 95% CI (bars) in relation to average provincial pig density per hectare (line), (c) Pooled provincial ribotype 078 CDI incidence rate and 95% CI (bars) in relation to average provincial pig density per hectare (line). DR: Drenthe, FL: Flevoland, FR: Friesland, GE: Gelderland, GR: Groningen, LI: Limburg, NB: North Brabant, NH: North Holland, OV: Overijssel, UT: Utrecht, ZE: Zeeland, ZH: South Holland.

Prospective national *C*. *difficile* infection sentinel surveillance (SeS) was initiated in the Netherlands in May 2009 by the National Reference Laboratory for *C*. *difficile* (Leiden University Medical Centre, Leiden and the National Institute for Public Health and the Environment (RIVM), Bilthoven). Hospitals were included in SeS according to their geographical location, with the aim to obtain a geographically representative sample of all hospitals in the Netherlands (Fig 1a). SeS hospitals prospectively submitted anonymous patient forms of all included CDI episodes to a web-based system (ethics approval not required). Subsequently, the National Reference Laboratory for *C*. *difficile* received the samples (faeces, or *C*. *difficile* isolates) of CDI episodes included in SeS for PCR ribotyping [19], and performed data analysis. For the current study, CDI SeS data from May 2009 to May 2015 was used. In that time period, surveillance was conducted at 26 hospitals (8/75 primary care hospitals, 12/23 secondary care hospitals, and 6/8 university hospitals), representing 25% of all hospitals in the Netherlands [14].

### Data collection and definitions

Hospitalised CDI patients (>2yrs of age) were eligible to be included in SeS. Children aged <2 yrs were excluded for reasons described earlier [20]. A case was defined as the presence of clinical symptoms (an abnormal stool frequency, >3 times per day diarrhoea during two subsequent days, or radiological or clinical signs of a toxic megacolon), and either the presence of a toxin-producing *C*. *difficile* in the faeces, or a confirmed pseudomembranous colitis (by endoscopy or by histopathology after colectomy or autopsy) [20, 21]. Participating hospitals applied their own diagnostic procedures to test for the presence of toxin-producing *C*. *difficile* in the faeces.

The patient form included information on the location of CDI onset (i.e. community or healthcare), the presence of CDI in the prior 8 weeks (‘recurrence’), CDI severity, CDI-related and all-cause 30-day mortality, as has been described previously [20]. Community-onset CDI included all cases with a reported onset of symptoms in the community, whereas healthcare-onset CDI related to cases with a reported onset of symptoms in a hospital, nursing home or other healthcare institution (e.g. care home). Four-digit postal code data was requested from patients with community-onset CDI. Severe CDI was defined by the presence of bloody diarrhoea and/or pseudomembranous colitis and/or diarrhoea with either dehydration and/or hypoalbuminemia, and/or fever (>38°C) and leucocytosis (>15.0 X 10^9^/L). 30-day mortality was considered ‘CDI-related’ if other comorbidities would not have caused death.

### Hospital incidence rates

Yearly hospital incidence rates of CDI were calculated per 10,000 inpatient-days [22]. Numbers of inpatient-days of participating hospitals were extracted from a website of the CIBG of the ministry of Health, Welfare and Sport, to ensure standardised data collection [23]. Children below the age of two could not be excluded from these denominator data, which was not considered to have a large impact on incidence rates of CDI. We calculated Poisson rate 95% CIs for all incidence rates. Overall and provincial incidence rate of CDI were generated by inverse-variance weighting.

### Provincial pig and piglet density

The StatLine database of the Dutch National Bureau of Statistics [14] was used to calculate four indicators of provincial pig-farming; (i) the number of pigs per hectare (‘pig density’), (ii) the number of piglets per hectare (‘piglet density’), (iii) the number of pig farms per 1000 hectare (‘pig farm density’), and (iv) the number of piglet farms per 1000 hectare (‘piglet farm density’) for 2009–2015.

### Statistical analysis

Risk ratios (RR) and 95% confidence intervals (CI) were calculated to compare patient characteristics of ribotype 078 CDI, with CDI caused by other ribotypes. Highly genetically related ribotypes that were difficult to discriminate by PCR ribotyping e.g. ribotypes 078/126 (as of now referred as ‘ribotype 078’) were clustered.

The association between four indicators of pig-farming (see Provincial pig and piglet density) and ribotype 078 hospital incidence rates at a provincial level was analysed by a multilevel mixed-effects Poisson regression model. We considered the outcome (incidence rate) to follow a Poisson distribution, and included ‘hospital’ as a random effect to account for clustered data. We calculated incidence rate ratios (IRR) for each of the four indicators of provincial pig-farming, and adjusted for diagnostic testing and year. Diagnostic testing was categorised into algorithms with ‘free toxin detection’, ‘PCR’ or ‘glutamate dehydrogenase detection’ since a variety of diagnostic algorithms were applied, and these categories are indicative for the sensitivity diagnostic testing for CDI. A similar analysis was performed for the hospital incidence of community-onset ribotype 078 infections. We performed two sensitivity analyses by (1) excluding university hospitals, where relatively more patients are treated that originate from a different province to receive highly specialised care, and (2) excluding ribotype 126 CDI from the ribotype 078 subgroup, to avoid potential misclassification. STATA software version 12.1 (StataCorp, College Station, USA) was used for data analysis.

### Spatial cluster analysis of community-onset CDI

To identify clusters of community-onset CDI that might be missed while investigating overall hospital incidence rates of CDI, we studied SeS postal code data of community-onset CDI by a retrospective space-time permutation model (SaTScan, M Kulldorff, Boston, MA, USA) [24]. This model does not require population-at-risk data, but data should derive from a stable population [24]. Therefore, we analysed data of two time periods (period I: September 2009-December 2013 and period II: December 2013-May 2015) of hospitals that continually participated in surveillance. To select an appropriate spatial window setting, we initially used 50% population-at-risk as maximal spatial cluster size, and repeated the analysis with a maximal cluster size of 25 km radius, and compared our results [25]. We searched for clusters of community-onset CDI in general, as well as for ribotype 078 specific clusters, and assessed if they were located in provinces with a high pig and piglet density. We performed a sensitivity analyses by excluding ribotype 126 CDI from the ribotype 078 subgroup, to avoid potential misclassification.

## Results

### Reported CDI episodes

In total, 4,691 CDI cases were reported by 26 hospitals in a period of six years. A third (n = 1,554) was designated as community-onset and two-thirds as healthcare-onset CDI (n = 3,038). Of all healthcare-onset CDI episodes, 2,751 (90.6%) were reported to have started in a hospital, 148 in a long-term care facility (4.9%) and 139 (4.6%) in other healthcare facilities. Healthcare-onset CDI was severe in 17.6% (n = 489) versus community-onset CDI in 34.9% (n = 517). Of patients with healthcare-onset CDI, 17.0% experienced a complicated course; 1 (0.04%) needed surgery for CDI, 37 (1.5%) had to be admitted to an intensive care unit for CDI, and 381 (15.5%) died within 30 days. Eight of these patients died due to CDI (0.3% of all HO-CDI), another 82 patients died from factors contributed to by CDI (3.3% of all HO-CDI). Of patients with community-onset CDI 11.3% experienced a complicated course; 8 (0.6%) needed surgery, 16 (1.2%) were admitted to an intensive care unit for CDI, and 126 (9.5%) died within 30 days. Two of these patients died due to CDI (0.15% of all CO-CDI) and 41 (3.1% of all CO-CDI) patients died from factors contributed to by CDI. Of the remaining patients cause of death was indeterminate, not related to CDI or unknown. S1 Table illustrates time trends of the characteristics and the outcome of all reported CDI episodes.

### Molecular typing of reported CDI episodes

For 3,755 CDI cases (80.0%) a PCR ribotyping result could be obtained and linked to the clinical data. Ribotype 014 (including ribotypes 020/295) was the most frequently isolated ribotype (n = 570; 15.2%), followed by ribotype 001 (n = 547; 14.6%). The occurrence of ribotype 001 declined in time (S1 Table). Ribotype 078 (including ribotype 126) was the third most commonly found ribotype (n = 493; 13.1%). Its prevalence was constant over the study period. Ribotype 027 was occasionally found (n = 97; 2.6%).

### CDI due to ribotype 078 compared to other ribotypes

The mean age of patients with a ribotype 078 infection was similar to those infected by other ribotypes (69 vs.67 years), but the age distribution was marginally different (*P* = 0.039; S2 Table). Community-onset CDI was slightly more common in 078 patients than in non-078 patients (RR, 1.13; 95% CI, 0.99–1.28). CDI severity was higher in 078 cases compared to cases caused by other ribotypes (RR, 1.28; 95% CI, 1.10–1.49). Further, patients with a ribotype 078 infection more often had a complicated course of disease (18.6% vs. 14.9%; RR, 1.25; 95% CI 1.00–1.56), a higher mortality (17.6 vs. 13.0%; RR, 1.35; 95% CI, 1.07–1.71), and higher CDI-related mortality (5.6 vs. 3.4%; RR, 1.65; 95% CI, 1.05–2.60) compared to those with CDI caused by other ribotypes.

### CDI incidence rates

**Fig 2:**
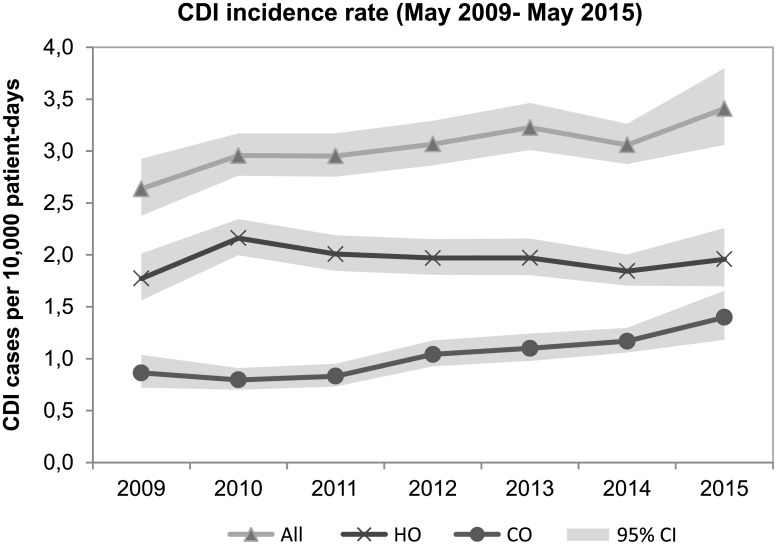
Incidence rate and 95% confidence interval of community-onset, hospital-onset and all CDI in participating hospitals (n = 26, 2009–2015). HO: hospital-onset, CO: community-onset.

The pooled overall and ribotype 078 specific CDI incidence rates were 2.82 (95% CI, 2.42–3.29) and 0.26 (95% CI, 0.21–0.31) cases per 10,000 patients-days respectively. There was no significant increase in time for all CDI (IRR, 1.00; 95% CI, 0.94–1.07) or ribotype 078 CDI (IRR, 1.04; 95% CI, 0.84–1.29) adjusted for diagnostic category. Community-onset CDI increased in time (IRR, 1.03; 95% CI 0.93–1.15) while hospital-onset CDI remained stable (IRR 0.98; 95% CI 0.90–1.06) when adjusting for diagnostic category (Fig 2). The overall CDI incidence rates were higher in secondary care hospitals (IRR, 1.44; 95% CI, 1.09–1.92) compared to primary care and university hospitals, as well as for ribotype 078 (IRR 1.44; 95% CI, 0.78–2.67) when adjusting for year and diagnostic category.

### CDI ribotype 078 incidence rates in relation to pig density

Fig 1b illustrates the pooled CDI incidence for each province with the corresponding average provincial pig density and Fig 1c the pooled provincial 078 incidence with the corresponding average pig density. Hospital ribotype 078 incidence was not associated pig density (IRR, 0.98; 95% CI, 0.88–1.09) or piglet density (IRR, 0.95; 95% CI, 0.75–1.21) of the province where the hospital was located. Further, ribotype 078 incidence was not associated with the number of pig farms per 1000 hectare (IRR, 1.08; 95% CI, 0.84–1.39) and the number of piglet farms per 1000 hectare (IRR, 1.00; 95% CI, 0.56–1.79). The incidence of community-onset ribotype 078 CDI was not related to the annual pig density (IRR, 0.98; 95% CI, 0.82–1.16) as well. The first sensitivity analysis excluding university hospitals (IRR, 0.98; 95% CI, 0.87–1.10) confirmed our primary results. The second sensitivity analysis excluding ribotype 126 from the ribotype 078 subgroup (IRR, 0.99; 95% CI, 0.89–1.10) also supported our primary findings.

### Spatial analysis of community-onset 078 clusters

Of patients with community-onset CDI (n = 1,554) the postal code was registered. For period I postal codes of 792 CO-CDIs (of which n = 90 ribotype 078) were analysed. For period II postal codes of 490 CO-CDIs (of which n = 52 ribotype 078) were included. In the analysis restricted to 25 km radius three large clusters (28, 37 and 49 km radius) of community-onset CDI were missed and five extra small clusters were detected (2, 5, 7, 8 and 20 km radius). None of these clusters were found to be statistically significant. We continued with the restricted model to detect smaller clusters that might be overlapped or non-significant in non-restricted settings [25]. In both time periods no clusters of community-onset CDI or community-onset ribotype 078 CDI were found. Also, in our sensitivity analysis (excluding ribotype 126 infections from the 078 subgroup) no significant clusters were detected.

## Discussion

*C*. *difficile* ribotype 078 persists as one of the most common ribotypes in hospitalised patients in the Netherlands, causing 13.1% of the cases in the present study. CDI due to type 078 was found to be associated with a worse clinical outcome (35% increase of 30-day mortality and 65% increase of CDI-related mortality) as in other studies [26]. We investigated the association between the hospital incidence of ribotype 078 CDI and pig farming at a provincial level as suggested before [16]. However, our results did not indicate any association of ribotype 078 CDI incidence in hospitals with provincial pig (farm) or piglet (farm) density. Besides, ribotype 078 did not cause spatial clusters of community-onset CDI followed by hospitalisation. Consequently we presume that *C*. *difficile* ribotype 078 shedding by pigs and/or piglets in our country does not lead to provincial excesses or localised clusters of hospital ribotype 078 CDI.

Earlier reports showed an association of ribotype 078 with community-onset or community-acquired disease [15–18] as one would expect if transmission was driven by pig or other animal contact in the community. We found ribotype 078 patients to have slightly higher community-onset disease compared to other patients. This could have been a result of the relatively low abundance of other endemic CDI strains (such as the healthcare-associated ribotype 027) taken as a reference to study ribotype 078 characteristics. Moreover, widespread transmission in the population may result in a change in clinical manifestation like for livestock-associated MRSA [27]. Previous studies in the Netherlands show that ribotype 078 was found in 11% of the CDI patients visiting their general practitioner with diarrhoea (absolute prevalence 0.09%) [28] and in 13% of the healthy community residents living in the proximity of livestock farms (absolute prevalence 0.16%) [29] similar to our results of hospitalised patients. In asymptomatic *C*. *difficile* carriers admitted to three Dutch hospitals (one hospital located in the province with the highest pig density, North Brabant), ribotype 078 was not one of the foremost ribotypes found [30]. These findings challenge the hypothesis that ribotype 078 primarily is a community-related disease.

This study is the first to search for clusters of community-onset ribotype 078 followed by hospitalisation in relation to animal density, as has been done for other stock-related pathogens such as Q-fever [25]. No significant clusters were found, in line with the absence of community outbreak reports of ribotype 078 CDI [5]. Remarkably, ribotype 078 rarely causes outbreaks in healthcare facilities (including those participating in the current study) despite its high virulence in infected patients.

### Limitations

Our study has several limitations. This surveillance study targeted a geographically representative sample of hospitals, but selection bias may nonetheless have occurred. Not all pig farming areas were included in our data. Our analysis was based on the assumption that *C*. *difficile* ribotype 078 shedding by pigs and piglets leads to enhanced human exposure in the province [13]. Exposure may occur more localised, but we did not have data on individual exposure to pig-farming in this study. Ribotype 078 CDI not associated or followed by hospitalisation were not included. Besides, our provincial indicators for pig-farming did not differentiate between areas of low and high intensive pig-farming. We only differentiated between community-onset and healthcare-onset CDI for feasibility, but community-onset CDI could be related to previous healthcare exposure. Therefore, our results probably reflect an overestimation of CDI that is actually acquired in the community. Diagnostics for CDI were not standardised in the Netherlands during this study, which could bias incidence rates. We adjusted for three diagnostic categories in our models, but not for the specific testing strategy and diagnostic algorithm. Currently, 87% of the participants in the sentinel surveillance study apply a two-step algorithm to diagnose CDI [31]. CDI incidence rates were slightly underestimated due to the fact that children below the age of two could not be excluded from denominator data. The space-time permutation model was hampered by a variable number of participating hospitals which would induce population shift bias. To avoid this, we split the data in two time periods and selected hospitals that continually participated in surveillance (∼80% all data) and thus might have missed clusters in the residual ∼20% of the data.

### Conclusions

According to the results of this study hospital incidence rates of ribotype 078 CDI in the Netherlands were not associated with pig-farming at a provincial level. Transmission beyond provincial borders (e.g. meat consumption) or in non-hospitalised patients cannot be excluded. No clusters of community-onset ribotype 078 followed by hospitalisation were detected in provinces with higher pig densities, although we might be scratching the surface of the burden of CDI in the community. For prospective studies on the zoonotic potential of CDI, we suggest that multiple reservoir hosts and ‘sinks’ of CDI are considered and advanced molecular methods are used to prove transmission.

## Supporting information

S1 Table Changes in the number of participating hospitals, the number of reported episodes, and the clinical characteristics and PCR ribotypes of all CDIs reported in *C*. *difficile* infection sentinel surveillance between May 2009 and May 2015. (DOCX)

S2 Table Patient characteristics of PCR ribotype 078 CDIs compared to non-078 CDIs reported in *C*. *difficile* infection sentinel surveillance between May 2009 and May 2015. (DOCX)

## Reporting

This study was reported according to the STROBE guidelines.
