# Supplementary material for: Ribotype 078 Clostridium difficile infection incidence in Dutch hospitals is not associated with provincial pig farming: Results from a national sentinel surveillance, 2009-2015
Source: PLoS One. 2017 Dec 29;12(12):e0189183. doi: 10.1371/journal.pone.0189183 (PMC5747436; doi:10.1371/journal.pone.0189183)
Supplement: S1 Table — (DOCX) [file pone.0189183.s001.docx]

|  | **2009/10** | | **2010/11** | | **2011/12** | | **2012/13** | | **2013/14** | | **2014/15** | | **Total** | |
| --- | --- | --- | --- | --- | --- | --- | --- | --- | --- | --- | --- | --- | --- | --- |
|  | ***No./%*** | ***(95% CI)*** | ***No./%*** | ***(95% CI)*** | ***No./%*** | ***(95% CI)*** | ***No./%*** | ***(95% CI)*** | ***No./%*** | ***(95% CI)*** | ***No./%*** | ***(95% CI)*** | ***No./%*** | ***(95% CI)*** |
| No. of participating hospitals | 19 | - | 20 | - | 18 | - | 19 | - | 21 | - | 22 | - | 26 |  |
| No. of reported CDI episodes | 677 | - | 750 | - | 748 | - | 750 | - | 825 | - | 941 | - | 4691 |  |
| Mean age in years | 68 | (66.5-69.3) | 67 | (65.5-68.2) | 66 | (64.2-67.1) | 67 | (65.5-68.2) | 67 | (65.7-68.3) | 67 | (66.1-68.5) | 66.9 | (66.4-67.5) |
| Females | 51% | (47.3-54.9%) | 49% | (45.8-52.9) | 52% | (48.5-55.7) | 52% | (48.8-56.0) | 51% | (47.9-54.7) | 51% | (48.2-54.6) | 51.3% | (49.8-52.7) |
| Community-onset CDI | 30% | (26.1-33.0) | 27% | (23.4-29.9) | 31% | (27.4-34.1) | 36% | (32.9-39.9) | 36% | (33.0-39.7) | 41% | (37.7-44.1) | 33.8% | (32.5-35.2) |
| Recurrent CDI | 33% | (27.9-38.2) | 22% | (18.0-25.5) | 24% | (19.8-27.6) | 23% | (19.1-27.6) | 24% | (20.4-27.8) | 23% | (19.5-26.2) | 24.3% | (22.7-25.9) |
| Severe CDI | 24% | (21.1-27.5) | 20% | (17.3-23.2) | 27% | (24.0-30.7) | 26% | (22.8-29.4) | 20% | (17.3-23.2) | 23% | (20.6-26.4) | 23.6% | (22.3-24.8) |
| Complicated course within 30 days | 20% | (17.1-23.7) | 15% | (12.3-18.0) | 15% | (11.9-17.6) | 14% | (11.2-16.7) | 13% | (10.6-15.7) | 14% | (11.3-16.2) | 15.1% | (13.9-16.2) |
| All-cause 30-day mortality | 18% | (15.1-21.5) | 14% | (10.8-16.2) | 14% | (10.9-16.4) | 12% | (9.8-15.1) | 11% | (8.5-13.3) | 13% | (10.2-15.0) | 13.4% | (12.3-14.5) |
| CDI-related 30-day mortality | 4% | (2.7-6.1) | 4% | (2.1-5.1) | 3% | (1.8-4.6) | 3% | (1.6-4.3) | 3% | (1.5-4.0) | 4% | (2.7-5.5) | 3.5% | (2.9-4.1) |
| Ribotype  001 | 27% | (22.8-30.5) | 21% | (17.6-24.2) | 15% | (12.3-17.9) | 14% | (11.3-16.8) | 9% | (7.1-11.6) | 6% | (4.2-7.6) | 14.6% | (13.4-15.7) |
| 014/020/295 | 12% | (9.0-14.6) | 14% | (11.5-17.1) | 16% | (12.7-18.5) | 17% | (13.6-19.4) | 15% | (12.3-17.8) | 17% | (14.2-19.6) | 15.2% | (14.0-16.3) |
| 078/126 | 11% | (8.7-14.1) | 14% | (10.8-16.4) | 15% | (12.0-17.6) | 13% | (10.7-16.1) | 12% | (9.9-15.0) | 13% | (10.6-15.4) | 13.1% | (12.0-14.2) |
| 027 | 4% | (2.5-6.0) | 2% | (1.1-3.6) | 2% | (1.1-3.5) | 3% | (2.0-4.8) | 3% | (1.9-4.6) | 1% | (0.1-1.2) | 2.6% | (2.1-3.1) |
| Other | 46% | (41.7-50.3) | 49% | (44.8-52.9) | 52% | (48.3-56.2) | 53% | (48.6-56.5) | 60% | (56.2-63.7) | 64% | (60.1-66.9) | 54.5% | (52.9-56.1) |
