# Supplementary material for: Ribotype 078 Clostridium difficile infection incidence in Dutch hospitals is not associated with provincial pig farming: Results from a national sentinel surveillance, 2009-2015
Source: PLoS One. 2017 Dec 29;12(12):e0189183. doi: 10.1371/journal.pone.0189183 (PMC5747436; doi:10.1371/journal.pone.0189183)
Supplement: S2 Table — (DOCX) [file pone.0189183.s002.docx]

|  | **078 CDI**  **(n=493)** | | **Non-078 CDI**  **(n=3,263)** | | **Risk ratio or P-value** | |
| --- | --- | --- | --- | --- | --- | --- |
|  | ***%*** | ***(95% CI)*** | ***%*** | ***(95% CI)*** | ***RR*** | ***(95% CI)*** |
| *Age* |  |  |  |  |  |  |
| < 18 years | 1.2% | (0.2-2.2) | 2.9% | (2.3-3.4) | *P* = .039 | |
| 18-65 years | 30.8% | (26.7-34.8) | 32.0% | (30.4-33.6) |  |  |
| 65-85 years | 56.4% | (52.0-60.8) | 51.2% | (49.5-53.0) |  |  |
| > 85 years | 11.6% | (8.8-14.4) | 13.9% | (12.7-15.1) |  |  |
| Female gender | 50.7% | (46.3-55.1) | 51.2% | (49.4-52.9) | 0.99 | (0.90-1.09) |
| *Hospital service* |  |  |  |  |  |  |
| Medical | 68.8% | (63.2-74.3) | 71.6% | (69.6-73.7) | *P* = .517 | |
| ICU | 5.5% | (2.8-8.2) | 4.3% | (3.4-5.2) |  |  |
| Surgery | 25.7% | (20.5-30.9) | 24.1% | (22.1-26.0) |  |  |
| Previous CDI (>8 weeks) | 29.6% | (24.3-35.0) | 23.1% | (21.2-25.0) | 1.28 | (1.05-1.57) |
| Community-onset of symptoms | 37.3% | (32.9-41.6) | 33.1% | (31.5-34.7) | 1.13 | (0.99-1.28) |
| Antibiotic therapy prior to CDI | 71.1% | (66.7-75.5) | 71.2% | (69.5-72.9) | 1.00 | (0.93-1.07) |
| Severe CDI | 30.3% | (26.1-34.6) | 23.7% | (22.2-25.3) | 1.28 | (1.10-1.49) |
